# Supplementary material for: Using ERIC to Assess Implementation Science in Drowning Prevention Interventions in High-Income Countries: A Systematic Review
Source: Int J Environ Res Public Health. 2023 Dec 27;21(1):45. doi: 10.3390/ijerph21010045 (PMC10815182; doi:10.3390/ijerph21010045)
Supplement: Supplementary file 1 [file ijerph-21-00045-s001.zip › ijerph-2686110-supplementary.pdf]

Supplementary Table S1: Systematic review search strategy (October 2022)

SEARCH ENGINE	SEARCH KEYWORDS	HITS^
Medline	(implement* or disseminat* or quality or evidence* or methodolog* or success* or translat*) AND (Implementation Science/ Evidence-Based Practice/ Program Evaluation/)	> 4 million
	(drown* and (prevent* or reduc*)) OR (drowning or near drowning/)	2477
	(implement* or disseminat* or quality or evidence* or methodolog* or success* or translat*) and (Implementation Science/ Evidence-Based Practice/ Program Evaluation/) AND (drown* and (prevent* or reduc*)) OR (drowning or near drowning/)	606
PubMed	(implement* or disseminat* or quality or evidence* or methodolog* or success* or translat*)	> 5 million
	(drown* and (prevent* or reduc*))	2303
	(implement* or disseminat* or quality or evidence* or methodolog* or success* or translat*) AND (drown* and (prevent* or reduc*))	464
Global health	(implement* or disseminat* or quality or evidence* or methodolog* or success* or translat*) AND (Implementation Science/ Evidence-Based Practice/ Program Evaluation/)	> 1 million
	(drown* and (prevent* or reduc*)) OR (drowning or near drowning/)	541
	(implement* or disseminat* or quality or evidence* or methodolog* or success* or translat*) and (Implementation Science/ Evidence-Based Practice/ Program Evaluation/) AND (drown* and (prevent* or reduc*)) OR (drowning or near drowning/)	194
ProQuest	noft (drown* and (prevent* or reduc*)) AND noft ((implement* or disseminat* or quality or evidence* or methodolog* or success* or translat*)	1058
	noft (drown* NEAR/5 (prevent* or reduc*)) AND noft ((implement* or disseminat* or quality or evidence* or methodolog* or success* or translat*)	344
PsycINFO	(implement* or disseminat* or quality or evidence* or methodolog* or success* or translat*) AND (Implementation Science/ Evidence-Based Practice/ Program Evaluation/)	> 1 million
	(drown* and (prevent* or reduc*)) OR (drowning or near drowning/)	262
	(implement* or disseminat* or quality or evidence* or methodolog* or success* or translat*) and (Implementation Science/ Evidence-Based Practice/ Program Evaluation/) AND (drown* and (prevent* or reduc*)) OR (drowning or near drowning/)	88
Web of Science	(implement* or disseminat* or quality or evidence* or methodolog* or success* or translat*)	> 15 million
	(drown* and (prevent* or reduc*)) OR (drowning or near drowning/)	3487
	(implement* or disseminat* or quality or evidence* or methodolog* or success* or translat*) AND (drown* and (prevent* or reduc*))	894
Scopus	(implement* or disseminat* or quality or evidence* or methodolog* or success* or translat*)	> 24 million
	(drown* and (prevent* or reduc*))	2661
	(implement* or disseminat* or quality or evidence* or methodolog* or success* or translat*) AND (drown* and (prevent* or reduc*))	901
SPORTDiscus	(implement* or disseminat* or quality or evidence* or methodolog* or success* or translat*)	160451
	(drown* and (prevent* or reduc*))	194
	(implement* or disseminat* or quality or evidence* or methodolog* or success* or translat*) AND (drown* and (prevent* or reduc*))	57

^Database search undertaken October 2022

Supplementary Table S2: Prisma Checklist Abstract

**Section and Topic**	**Item #**	**Checklist item**	**Reported (Yes/No)**
**TITLE**			
Title	1	Identify the report as a systematic review.	yes
**BACKGROUND**			
Objectives	2	Provide an explicit statement of the main objective(s) or question(s) the review addresses.	yes
**METHODS**			
Eligibility criteria	3	Specify the inclusion and exclusion criteria for the review.	yes
Information sources	4	Specify the information sources (e.g. databases, registers) used to identify studies and the date when each was last searched.	yes
Risk of bias	5	Specify the methods used to assess risk of bias in the included studies.	no
Synthesis of results	6	Specify the methods used to present and synthesise results.	yes
**RESULTS**			
Included studies	7	Give the total number of included studies and participants and summarise relevant characteristics of studies.	yes
Synthesis of results	8	Present results for main outcomes, preferably indicating the number of included studies and participants for each. If meta-analysis was done, report the summary estimate and confidence/credible interval. If comparing groups, indicate the direction of the effect (i.e. which group is favoured).	yes
**DISCUSSION**			
Limitations of evidence	9	Provide a brief summary of the limitations of the evidence included in the review (e.g. study risk of bias, inconsistency and imprecision).	no
Interpretation	10	Provide a general interpretation of the results and important implications.	yes
**OTHER**			
Funding	11	Specify the primary source of funding for the review.	no
Registration	12	Provide the register name and registration number.	yes

From: Page MJ, McKenzie JE, Bossuyt PM, Boutron I, Hoffmann TC, Mulrow CD, et al. The PRISMA 2020 statement: an updated guideline for reporting systematic reviews. BMJ 2021;372:n71. doi: 10.1136/bmj.n71

Supplementary Table S3: Prisma Checklist Manuscript

Section and Topic	Item Number	Checklist item	Location where item is reported
**TITLE**			
Title	1	Identify the report as a systematic review.	Page 1
**ABSTRACT**			
Abstract	2	See the PRISMA 2020 for Abstracts checklist.	Supplementary Table 2
**INTRODUCTION**			
Rationale	3	Describe the rationale for the review in the context of existing knowledge.	Page 1 - 3
Objectives	4	Provide an explicit statement of the objective(s) or question(s) the review addresses.	Page 3
**METHODS**			
Eligibility criteria	5	Specify the inclusion and exclusion criteria for the review and how studies were grouped for the syntheses.	Page 3 & Table 1
Information sources	6	Specify all databases, registers, websites, organisations, reference lists and other sources searched or consulted to identify studies. Specify the date when each source was last searched or consulted.	Page 4 and Supplementary table 1
Search strategy	7	Present the full search strategies for all databases, registers and websites, including any filters and limits used.	Supplementary table 1
Selection process	8	Specify the methods used to decide whether a study met the inclusion criteria of the review, including how many reviewers screened each record and each report retrieved, whether they worked independently, and if applicable, details of automation tools used in the process.	Page 3 -5
Data collection process	9	Specify the methods used to collect data from reports, including how many reviewers collected data from each report, whether they worked independently, any processes for obtaining or confirming data from study investigators, and if applicable, details of automation tools used in the process.	Page 4
Data items	10a	List and define all outcomes for which data were sought. Specify whether all results that were compatible with each outcome domain in each study were sought (e.g. for all measures, time points, analyses), and if not, the methods used to decide which results to collect.	Page 5
	10b	List and define all other variables for which data were sought (e.g. participant and intervention characteristics, funding sources). Describe any assumptions made about any missing or unclear information.	Page 5
Study risk of bias assessment	11	Specify the methods used to assess risk of bias in the included studies, including details of the tool(s) used, how many reviewers assessed each study and whether they worked independently, and if applicable, details of automation tools used in the process.	Page 4 -5
Effect	12	Specify for each outcome the effect measure(s) (e.g. risk	Not included

Section and Topic	Item Number	Checklist item	Location where item is reported
measures		ratio, mean difference) used in the synthesis or presentation of results.	
Synthesis methods	13a	Describe the processes used to decide which studies were eligible for each synthesis (e.g. tabulating the study intervention characteristics and comparing against the planned groups for each synthesis (item #5)).	Page 4
	13b	Describe any methods required to prepare the data for presentation or synthesis, such as handling of missing summary statistics, or data conversions.	Footnote table 3
	13c	Describe any methods used to tabulate or visually display results of individual studies and syntheses.	Page 5
	13d	Describe any methods used to synthesize results and provide a rationale for the choice(s). If meta-analysis was performed, describe the model(s), method(s) to identify the presence and extent of statistical heterogeneity, and software package(s) used.	Not included
	13e	Describe any methods used to explore possible causes of heterogeneity among study results (e.g. subgroup analysis, meta-regression).	Not included
	13f	Describe any sensitivity analyses conducted to assess robustness of the synthesized results.	Not included
Reporting bias assessment	14	Describe any methods used to assess risk of bias due to missing results in a synthesis (arising from reporting biases).	Not included
Certainty assessment	15	Describe any methods used to assess certainty (or confidence) in the body of evidence for an outcome.	Not included
**RESULTS**			
Study selection	16a	Describe the results of the search and selection process, from the number of records identified in the search to the number of studies included in the review, ideally using a flow diagram.	Page 4
	16b	Cite studies that might appear to meet the inclusion criteria, but which were excluded, and explain why they were excluded.	Page 4
Study characteristics	17	Cite each included study and present its characteristics.	Table 3
Risk of bias in studies	18	Present assessments of risk of bias for each included study.	Page 4
Results of individual studies	19	For all outcomes, present, for each study: (a) summary statistics for each group (where appropriate) and (b) an effect estimate and its precision (e.g. confidence/credible interval), ideally using structured tables or plots.	Table 4
Results of syntheses	20a	For each synthesis, briefly summarise the characteristics and risk of bias among contributing studies.	Table 4
	20b	Present results of all statistical syntheses conducted. If meta-analysis was done, present for each the summary estimate and its precision (e.g. confidence/credible interval)	Page 6 - 10

Section and Topic	Item Number	Checklist item	Location where item is reported
		and measures of statistical heterogeneity. If comparing groups, describe the direction of the effect.	
	20c	Present results of all investigations of possible causes of heterogeneity among study results.	Page 6 - 10
	20d	Present results of all sensitivity analyses conducted to assess the robustness of the synthesized results.	Not included
Reporting biases	21	Present assessments of risk of bias due to missing results (arising from reporting biases) for each synthesis assessed.	Page 13
Certainty of evidence	22	Present assessments of certainty (or confidence) in the body of evidence for each outcome assessed.	Not included
**DISCUSSION**			
Discussion	23a	Provide a general interpretation of the results in the context of other evidence.	Page 10 -13
	23b	Discuss any limitations of the evidence included in the review.	Page 10 -13
	23c	Discuss any limitations of the review processes used.	Page 13
	23d	Discuss implications of the results for practice, policy, and future research.	Page 12
**OTHER INFORMATION**			
Registration and protocol	24a	Provide registration information for the review, including register name and registration number, or state that the review was not registered.	Page 3
	24b	Indicate where the review protocol can be accessed, or state that a protocol was not prepared.	Page 3
	24c	Describe and explain any amendments to information provided at registration or in the protocol.	Not relevant
Support	25	Describe sources of financial or non-financial support for the review, and the role of the funders or sponsors in the review.	Acknowledgements
Competing interests	26	Declare any competing interests of review authors.	Conflict of interest
Availability of data, code and other materials	27	Report which of the following are publicly available and where they can be found: template data collection forms; data extracted from included studies; data used for all analyses; analytic code; any other materials used in the review.	All available from corresponding author

From: Page MJ, McKenzie JE, Bossuyt PM, Boutron I, Hoffmann TC, Mulrow CD, et al. The PRISMA 2020 statement: an updated guideline for reporting systematic reviews. BMJ 2021;372:n71. doi: 10.1136/bmj.n71

Supplementary Table S4: Refinement of the ERIC strategies

***ORIGINAL* WITH (COMMUNITY SETTING CHANGES)**		**THIS RESEARCH**		
ERIC CONCEPTS & STRATEGIES	DEFINITION	CONCEPTS & STRATEGIES	DEFINITION	CONCEPT & STRATEGY IDENTIFIER
CONCEPT 1: Use evaluative and iterative strategies		CONCEPT 1: Use evaluative and iterative strategies		c1
Assess for readiness and identify barriers and facilitators	Assess various aspects of an organization to determine its degree of readiness to implement, barriers that may impede implementation, and strengths that can be used in the implementation effort	Assess for readiness and identify barriers and facilitators	Assess various aspects of an organisation to determine its degree of readiness to implement, barriers that may impede implementation, and strengths that can be used in the implementation effort.	c1_01
Audit and provide feedback	Collect and summarize *clinical* performance data over a specified time period and give it to *clinicians* and administrators to monitor, evaluate, and modify provider behaviour.	Audit and provide feedback	Collect and summarize **intervention** performance data over a specified time period and give it to **providers** and administrators to monitor, evaluate, and modify provider behaviour.	c1_02
Conduct cyclical small tests of change	Implement changes in a cyclical fashion using small tests of change before taking changes system-wide. Tests of change benefit from systematic measurement, and results of the tests of change are studied for insights on how to do better. This process continues serially over time, and refinement is added with each cycle.	Conduct cyclical small tests of change	Implement changes in a cyclical fashion using small tests of change before taking changes system-wide. Tests of change benefit from systematic measurement, and results of the tests of change are studied for insights on how to do better. This process continues serially over time, and refinement is added with each cycle.	c1_03
Conduct local needs assessment	Collect and analyse data related to the need for the *innovation*	Conduct local needs assessment	Collect and analyse data related to the need for the **intervention (with the target group or their support network)**	c1_04
Develop a formal implementation blueprint	Develop a formal implementation blueprint that includes all goals and strategies. The blueprint should include the following: 1) aim/purpose of the implementation;<br>2) scope of the change (e.g., what organizational units are affected); 3) timeframe and milestones; and 4) appropriate performance/progress measures. Use and update this plan to guide the implementation effort over time.	Develop a formal implementation blueprint	Develop a formal implementation blueprint that includes all goals and strategies. The blueprint should include the following: 1) aim/purpose of the implementation; 2) scope of the change (e.g., what organizational units are affected); 3) timeframe and milestones; and 4) appropriate performance/progress measures. Use and update this plan to guide the implementation effort.	c1_05

ORIGINAL WITH (COMMUNITY SETTING CHANGES)		THIS RESEARCH		
ERIC CONCEPTS & STRATEGIES	DEFINITION	CONCEPTS & STRATEGIES	DEFINITION	CONCEPT & STRATEGY IDENTIFIER
Develop and implement tools for quality-monitoring	Develop, test, and introduce into quality-monitoring systems the right input—the appropriate language, protocols, algorithms, standards, and measures (of processes, patient/consumer outcomes, and implementation outcomes) that are often specific to the *innovation* being implemented	Develop and implement tools for quality-monitoring	Develop, test, and introduce into quality-monitoring systems the right input—the appropriate language, protocols, algorithms, standards, and measures (of processes, patient/consumer outcomes, and implementation outcomes) that are often specific to the **intervention** being implemented	c1_06
Develop and organize quality-monitoring systems	Develop and organize systems and procedures that monitor *clinical* processes and/or outcomes for the purpose of quality assurance and improvement.	Develop and organize quality-monitoring systems	Develop and organize systems and procedures that monitor intervention processes and/or outcomes for the purpose of quality assurance and improvement.	c1_07
Obtain and use *patients/consumers & family* (priority population & support network) feedback	Develop strategies to increase *patient/consumer and family* feedback on the implementation effort	Obtain and use target group & support network feedback	Develop strategies to increase **target group and support networks** feedback on the implementation effort	c1_08
Purposely re-examine the implementation	Monitor progress and adjust *clinical* practices and implementation strategies to continuously improve the quality of care	Purposely re-examine the implementation	Monitor progress and adjust **intervention** practices and implementation strategies to continuously improve the quality of the **intervention**	c1_09
Stage implementation scale-up	Phase implementation efforts by starting with small pilots or demonstration projects and gradually move to a system wide rollout	Stage implementation scale-up	Phase implementation efforts by starting with small pilots or demonstration projects and gradually move to a system wide rollout	c1_10
CONCEPT 2: Provide interactive assistance		CONCEPT 2: Provide interactive assistance		c2
Centralise technical assistance	Develop and use a centralized system to deliver technical assistance focused on implementation issues	*Centralise technical assistance	Develop and use a centralized system to deliver technical assistance focused on implementation issues	c2_01
Facilitation	A process of interactive problem solving and support that occurs in a context of a recognized need for improvement and a supportive interpersonal relationship	Facilitation	A process of interactive problem solving and support that occurs in a context of a recognized need for improvement and a supportive interpersonal relationship	c2_02

ORIGINAL WITH (COMMUNITY SETTING CHANGES)		THIS RESEARCH		
ERIC CONCEPTS & STRATEGIES	DEFINITION	CONCEPTS & STRATEGIES	DEFINITION	CONCEPT & STRATEGY IDENTIFIER
Provide technical assistance	Develop and use a system to deliver technical assistance focused on implementation issues using local personnel	Provide technical assistance	Develop and use a system to deliver technical assistance focused on implementation issues using local personnel	c2_03
Provide *clinical* supervision	Provide *clinicians* with ongoing supervision focusing on the *innovation*. Provide training for *clinical* supervisors who will supervise *clinicians* who provide the *innovation*	Provide supervision	Provide **providers** with ongoing supervision focusing on the **intervention**. Provide training for provider supervisors who will supervise **providers** of the **intervention**	c2_04
CONCEPT 3: Adapt and tailor context		CONCEPT 3: Adapt and tailor context		c3
Promote adaptability	Identify the ways a *clinical* innovation can be tailored to meet local needs and clarify which elements of the *innovation* must be maintained to preserve fidelity	Promote adaptability	Identify the ways an **intervention** can be tailored to meet local needs and clarify which elements of **the intervention** must be maintained to preserve fidelity	c3_01
Tailor strategies	Tailor the implementation strategies to address barriers and leverage facilitators that were identified through earlier data collection	Tailor strategies	Tailor the implementation strategies to address barriers and leverage facilitators that were identified through earlier data collection	c3_02
Use data experts	Involve, hire, and/or consult experts to inform management on the use of data generated by implementation efforts	*Use data experts	Involve, hire, and/or consult experts to inform management on the use of data generated by implementation efforts	c3_03
Use data warehouse techniques	Integrate *clinical* records across facilities and organizations to facilitate implementation across systems	*Use data warehouse techniques	Integrate **intervention** records across facilities and organizations to facilitate implementation across systems	c3_04
CONCEPT 4: Develop partner relationships		CONCEPT 4: Develop partner relationships		c4
Build a coalition	Recruit and cultivate relationships with partners in the implementation effort.	Build a coalition	Recruit and cultivate relationships with partners in the implementation effort **with partners involved throughout the whole intervention**	c4_01
Capture and share local knowledge	Capture local knowledge from implementation sites on how implementers and *clinicians* made something work in their setting and then share it with other sites	Capture and share local knowledge	Capture local knowledge from implementation sites on how implementers and **providers** made something work in their setting and then share it with other sites	c4_02

ORIGINAL WITH (COMMUNITY SETTING CHANGES)		THIS RESEARCH		
ERIC CONCEPTS & STRATEGIES	DEFINITION	CONCEPTS & STRATEGIES	DEFINITION	CONCEPT & STRATEGY IDENTIFIER
Conduct local consensus discussions	Collect and analyse data related to the need for the *innovation*	Conduct local consensus discussions	Collect and analyse data related to the need for the **innovation (done with stakeholders)**	c4_03
Develop academic partnerships	Partner with a university or academic unit for the purposes of shared training and bringing research skills to an implementation project	Develop academic partnerships	Partner with a university or academic unit for the purposes of shared training and bringing research skills to an implementation project	c4_04
Develop an implementation glossary	Develop and distribute a list of terms describing the *innovation*, implementation, and stakeholders in the organizational change	Develop an implementation glossary	Develop and distribute a list of terms describing the **intervention**, implementation, and stakeholders in the organisational change	c4_05
Identify and prepare champions	Identify and prepare individuals who dedicate themselves to supporting, marketing, and driving through an implementation, overcoming indifference or resistance that the intervention may provoke in an organization (intended community	Identify and prepare champions	Identify and prepare individuals who dedicate themselves to supporting, marketing, and driving through an implementation, overcoming indifference or resistance that the intervention may provoke in an organization **or** intended community	c4_06
Identify early adopters	Identify early adopters at the local site to learn from their experiences with the practice *innovation*	Identify early adopters	Identify early adopters at the local site to learn from their experiences with the practice **intervention**	c4_07
Inform local opinion leaders	Inform providers identified by colleagues as opinion leaders or “educationally influential” about the *clinical innovation* in the hopes that they will influence colleagues to adopt it	Inform local opinion leaders	Inform providers identified by colleagues as opinion leaders or “educationally influential” about the **intervention** in the hopes that they will influence colleagues to adopt it	c4_08
Involve executive boards	Involve existing governing structures (e.g., boards of directors, medical staff boards of governance) in the implementation effort, including the review of data on implementation processes	Involve executive boards	Involve existing governing structures (e.g., boards of directors, medical staff boards of governance) in the implementation effort, including the review of data on implementation processes	c4_09
Model and simulate change	Model or simulate the change that will be implemented prior to implementation	Model and simulate change	Model or simulate the change that will be implemented prior to implementation	c4_10
Obtain formal commitments	Obtain written commitments from key partners that state what they will do to implement the innovation	Obtain formal commitments	Obtain written commitments from key partners that state what they will do to implement the intervention	c4_11

ORIGINAL WITH (COMMUNITY SETTING CHANGES)		THIS RESEARCH		
ERIC CONCEPTS & STRATEGIES	DEFINITION	CONCEPTS & STRATEGIES	DEFINITION	CONCEPT & STRATEGY IDENTIFIER
Organise c*linician* implementation team meetings	Develop and support teams of *clinicians* who are implementing the *innovation* and give them protected time to reflect on the implementation effort, share lessons learned, and support one another's learning	Organise implementation team meetings	Develop and support teams of **providers** who are implementing the intervention and give them protected time to reflect on the implementation effort, share lessons learned, and support one another's learning	c4_12
Promote network *weavings*	Identify and build on existing high-quality working relationships and networks within and outside the organization, organizational units, teams, etc. to promote information sharing, collaborative problem-solving, and a shared vision/goal related to implementing the *innovation.*	Promote network **collaboration**	Identify and build on existing high-quality working relationships and networks within and outside the organization, organizational units, teams, etc. to promote information sharing, collaborative problem-solving, and a shared vision/goal related to implementing the **intervention**.	c4_13
Recruit, designate and train for leadership	Recruit, designate, and train leaders for the change effort	Recruit, designate and train for leadership	Recruit, designate, and train leaders for the change effort	c4_14
Use advisory boards and workgroups	Create and engage a formal group of multiple kinds of stakeholders to provide input and advice on implementation efforts and to elicit recommendations for improvements.	Use advisory boards and workgroups	Create and engage a formal group of multiple kinds of stakeholders to provide input and advice on implementation efforts and to elicit recommendations for improvements.	c4_15
Use an implementation advisor	Seek guidance from experts in implementation	Use an implementation advisor	Seek guidance from experts in implementation	c4_16
Visit other sites	Visit sites where a similar implementation effort has been considered successful	Visit other sites	Visit sites where a similar implementation effort has been considered successful	c4_17
CONCEPT 5: Train and educate *collaborators*		CONCEPT 5: Train and educate **stakeholders**		c5
Conduct educational meetings	Hold meetings targeted toward different stakeholder groups (e.g., providers, administrators, other organizational stakeholders, and community, *patient/consumer, and family* stakeholders) to teach them about the *clinical innovation*	Conduct educational meetings	Hold meetings targeted toward different stakeholder groups (e.g., providers, administrators, other organizational stakeholders, and **target group, and support network** stakeholders) to teach them about the **intervention**	c5_01

ORIGINAL WITH (COMMUNITY SETTING CHANGES)		**THIS RESEARCH**		
ERIC CONCEPTS & STRATEGIES	DEFINITION	CONCEPTS & STRATEGIES	DEFINITION	CONCEPT & STRATEGY IDENTIFIER
Conduct educational outreach visits	Have a trained person meet with providers in their *practice* settings to educate providers about *the clinical innovation* with the intent of changing the provider's practice	Conduct educational outreach visits	Have a trained person meet with providers in their **provider** settings to educate providers about the **intervention** with the intent of changing the provider's practice	c5_02
Conduct ongoing training	Plan for and conduct training in the *clinical innovation* in an ongoing way	Conduct ongoing training	Plan for and conduct training in **the intervention or implementation** in an ongoing way	c5_03
Create a learning collaborative	Facilitate the formation of groups of providers or provider organizations and foster a collaborative learning environment to improve implementation of the *clinical innovation*	Create a learning collaborative	Facilitate the formation of groups of providers or provider organisations and foster a collaborative learning environment to improve implementation of the **intervention**	c5_04
Develop educational materials	Develop and format manuals, toolkits, and other supporting materials in ways that make it easier for stakeholders to learn about the *innovation* and for *clinicians* to learn how to deliver the *clinical innovation*	Develop educational materials	Develop and format manuals, toolkits, and other supporting materials in ways that make it easier for stakeholders to learn about the **intervention** and for **providers** to learn how to deliver the **intervention**	c5_05
Distribute educational materials	Distribute educational materials (including guidelines, manuals, and toolkits) in person, by mail, and/or electronically	Distribute educational materials	Distribute educational materials (including guidelines, manuals, and toolkits) in person, by mail, and/or electronically	c5_06
Make training dynamic	Vary the information delivery methods to cater to different learning styles and work contexts, and shape the training in the *innovation* to be interactive	Make training dynamic	Vary the information delivery methods to cater to different learning styles and work contexts, and shape the training in the **intervention** to be interactive	c5_07
Provide ongoing consultation	Provide ongoing consultation with one or more experts in the strategies used to support implementing the *innovation*	Provide ongoing consultation	Provide ongoing consultation with one or more experts in the strategies used to support implementing the **intervention**	c5_08
Shadow other experts	Provide ways for key individuals to directly observe experienced *people* engage with or use the targeted *practice change/innovation*	Shadow other experts	Provide ways for key individuals to directly observe experienced **providers** engage with or use the targeted **intervention**	c5_09
Use train-the-trainer strategies	Train designated *clinicians* or organizations to train others in the *clinical innovation*	Use train-the-trainer strategies	Train designated **providers** or organizations to train others in the **intervention**	c5_10

***ORIGINAL* WITH (COMMUNITY SETTING CHANGES)**		**THIS RESEARCH**		
ERIC CONCEPTS & STRATEGIES	DEFINITION	CONCEPTS & STRATEGIES	DEFINITION	CONCEPT & STRATEGY IDENTIFIER
Work with educational institutions	Encourage educational institutions to train *clinicians* in the *innovation*	Work with educational institutions	Encourage educational institutions to train **providers** in the **intervention or implementation**	c5_11
CONCEPT 6: Support *clinicians (delivery agents)*		CONCEPT 6: Support **providers**		c6
Create new *clinical (delivery agent)* teams	Change who serves on the *clinical* team, adding different disciplines and different skills to make it more likely that the *clinical innovation* is delivered (or is more successfully delivered)	Create new provider teams	Change who serves on the **provider** team, adding different disciplines and different skills to make it more likely that the **intervention** is delivered (or is more successfully delivered)	c6_01
Develop resource sharing agreements	Develop partnerships with organizations that have resources needed to implement the *innovation*	Develop resource sharing agreements	Develop partnerships with organizations that have resources needed to implement the **intervention. Includes cases whereby existing resources were used but no sharing agreement was mentioned.**	c6_02
Facilitate relay of *clinical* data to providers (delivery agents)	Provide as close to real-time data as possible about key measures of process/outcomes using integrated modes/channels of communication in a way that promotes use of the targeted *innovation*	Facilitate relay of **program** data to providers	Provide as close to real-time data as possible about key measures of process/outcomes using integrated modes/channels of communication in a way that promotes use of the targeted **intervention**	c6_03
Reminder *clinicians (implementers (agents, volunteers, paraprofessionals))*	Develop reminder systems designed to help clinicians to recall information and/or prompt them to use the clinical innovation	Remind providers	Develop reminder systems designed to help clinicians to recall information and/or prompt them to use the clinical innovation	c6_04
Revise professional roles	Shift and revise roles among professionals who provide care, and redesign job characteristics	Revise professional roles	Shift and revise roles among professionals who provide care, and redesign job characteristics	c6_05
CONCEPT 7: Engage *consumers (priority populations)*		CONCEPT 7: Engage **target group**		c7
Increase demand	Attempt to influence the market for the *clinical innovation* to increase competition intensity and to increase the maturity of the market for the *clinical innovation*	Increase demand	Attempt to influence the market for the **intervention** to increase competition intensity and to increase the maturity of the market for the **intervention**	c7_01
Intervene with *patients/consumers and family members (priority population)*	Develop strategies *with patients* to encourage and problem solve around adherence	Intervene with **target group** to enhance uptake and adherence	Develop strategies with the **target group and/or their support network** to encourage and problem solve around adherence	c7_02

ORIGINAL WITH (COMMUNITY SETTING CHANGES)		THIS RESEARCH		
ERIC CONCEPTS & STRATEGIES	DEFINITION	CONCEPTS & STRATEGIES	DEFINITION	CONCEPT & STRATEGY IDENTIFIER
to enhance uptake and adherence				
Involve *patients /consumers and family* members *(priority population* and support network*)*	Engage or include *patients/consumers and families* in the implementation effort	Involve target group and support network	Engage or include the **target group and/or their support network** in the implementation effort	c7_03
Prepare *patient/consumers (priority population*) to actively participate	Prepare *patients/consumers* to be active in their *care*, to ask questions, and specifically to inquire about *care* guidelines, the evidence behind *clinical* decisions*, or about available evidence-supported treatments*	Prepare **target group** to actively participate	Prepare the **target group** to be active in their **involvement**, to ask questions, and specifically to inquire about guidelines and the evidence behind the **intervention**	c7_04
Use mass media	Use media to reach large numbers of people to spread the word about the *clinical innovation*	Use mass media	Use media to reach large numbers of people to spread the word about the **intervention**	c7_05
CONCEPT 8: Financial strategies		CONCEPT 8: Financial strategies		c8
Access new funding	Access new or existing money to facilitate the implementation	Access new funding	Access new or existing money to facilitate the implementation	c8_01
Alter incentives/allowance structures *for clinicians*	Work to incentivize the adoption and implementation of the *clinical innovation*	Alter **provider** incentives/allowance structures	Work to incentivize the adoption and implementation of the **intervention**	c8_02
Alter *patient/ consumer (end user)* fees	Create fee structures where *patients/consumers* pay less for preferred treatments (the clinical innovation) and more for less-preferred treatments	Alter fees for **target group**	Create fee structures where the target group or their support network pay less for the **intervention (eg. community pool access)**	c8_03
Develop disincentives	Provide financial disincentives for failure to implement or use the *clinical innovations*	Develop disincentives	Provide financial disincentives for failure to implement or use the **intervention**	c8_04
Fund and contract for the *clinical innovation (evidence-based programs)*	Governments and other payers of services issue requests for proposals to deliver the *innovation*, use contracting processes to motivate providers to deliver the *clinical innovation*, and develop	Fund and contract for the **evidence-informed intervention**	Governments and other payers of services issue requests for proposals to deliver the intervention, use contracting processes to motivate providers to deliver the **intervention**,	c8_05

ORIGINAL WITH (COMMUNITY SETTING CHANGES)		THIS RESEARCH		
ERIC CONCEPTS & STRATEGIES	DEFINITION	CONCEPTS & STRATEGIES	DEFINITION	CONCEPT & STRATEGY IDENTIFIER
	new funding formulas that make it more likely that providers will deliver the *innovation*		and develop new funding formulas that make it more likely that providers will deliver the **intervention**	
Make billing easier	Make it easier to bill for the *clinical innovation*	*Make billing easier	Make it easier to bill for the **intervention**	c8_06
Place *innovation (evidence-based programs)* on a fee-for-service list/formularies	Work to place the *clinical innovation* on lists of actions for which providers can be reimbursed *(e.g., a drug is placed on a formulary, a procedure is now reimbursable)*	Place **interventions** on a fee-for-service list/formularies	Work to place the **intervention** on lists of actions for which providers can be reimbursed **(e.g. pool fence compliance checks charged to pool owners)**	c8_07
Use capitated payments	Pay providers *or care systems* a set amount per *patient/consumer* for delivering *clinical care*	*Use capitated payments	Pay providers a set amount per **target group member** for delivering the **intervention**	c8_08
Use other payment schemes	Introduce payment approaches (in a catch-all category)	*Use other payment schemes	Introduce payment approaches (in a catch-all category)	c8_09
CONCEPT 9: Change infrastructure		CONCEPT 9: Change infrastructure		c9
Change accreditation or membership requirements	Strive to alter accreditation standards so that they require or encourage use of the *clinical innovation*. Work to alter membership organization requirements so that those who want to affiliate with the organization are encouraged or required to use the *clinical innovation*	*Change accreditation or membership requirements	Strive to alter accreditation standards so that they require or encourage use of the **intervention.** Work to alter membership organization requirements so that those who want to affiliate with the organization are encouraged or required to use the **intervention**	c9_01
Change liability laws	Participate in liability reform efforts that make *clinicians* more willing to deliver the *clinical innovation*	Change liability laws **or enforcement**	Participate in liability reform efforts that make **providers** more willing to deliver the **intervention**	c9_02
Change physical structure and equipment	Evaluate current configurations and adapt, as needed, the physical structure and/or equipment (e.g., changing the layout of a room, adding equipment) to best accommodate the targeted *innovation*	Change physical structure and equipment	Evaluate current configurations and adapt, as needed, the physical structure and/or equipment (e.g., changing the layout of a room, adding equipment) to best accommodate the targeted i**ntervention**	c9_03
Change record systems	Change records systems to allow better assessment of implementation or *clinical* outcomes	Change record systems	Change records systems to allow better assessment of implementation outcomes	c9_04

***ORIGINAL* WITH (COMMUNITY SETTING CHANGES)**		**THIS RESEARCH**		
ERIC CONCEPTS & STRATEGIES	DEFINITION	CONCEPTS & STRATEGIES	DEFINITION	CONCEPT & STRATEGY IDENTIFIER
Change service sites	Change the location of *clinical* service sites to increase access	Change service sites	Change the location of service sites to increase access	c9_05
Create or change credentialing and or licensure standards	Create an organization that certifies *clinicians* in the *innovation* or encourage an existing organization to do so. Change governmental professional certification or licensure requirements to include delivering the *innovation*. Work to alter continuing education requirements to shape professional practice toward the *innovation*	*Create or change credentialing and or licensure standards	Create an organization that certifies **providers** in the **intervention** or encourage an existing organization to do so. Change governmental professional certification or licensure requirements to include delivering the **intervention**. Work to alter continuing education requirements to shape professional practice toward the **intervention**	c9_06
Mandate change	Have leadership declare the priority of the *innovation* and their determination to have it implemented	Mandate change	Have leadership declare the priority of the **intervention** and their determination to have it implemented	c9_07
Start a dissemination organisation	Identify or start a separate organization that is responsible for disseminating the *clinical innovation*. It could be a for-profit or non-profit organization	Start a dissemination organisation	Identify or start a separate organization that is responsible for disseminating the **intervention**. It could be a for-profit or non-profit organization	c9_08

* indicates strategies of a technical/clinical nature, not expected to be seen in the literature
Italicised text indicates text removed from original ERIC strategies names and or definitions
Bold text indicates text added for this review
Bracketed text () indicates text added by Balis and colleagues to the original ERIC strategies

This table is adapted from previous work undertaken to develop and refine the Expert Recommendation for Implementing Change (ERIC) strategies. Columns 1 and 2 are adapted from

Powell, B. J., Waltz, T. J., Chinman, M. J., Damschroder, L. J., Smith, J. L., Matthieu, M. M., Proctor, E. K., & Kirchner, J. E. (2015). A refined compilation of implementation strategies: results from the Expert Recommendations fo Implementing Change (ERIC) project. Implementation Science, 10(1), 21. https://doi.org/10.1186/s13012-015-0209-1;

Waltz, T. J., Powell, B. J., Matthieu, M. M., Damschroder, L. J., Chinman, M. J., Smith, J. L., Proctor, E. K., & Kirchner, J. E. (2015). Use of concept mapping to characterize relationships among implementation strategies and assess their feasibility and importance: results from the Expert Recommendations for Implementing Change (ERIC) study. Implementation Science, 10(1). https://doi.org/10.1186/s13012-015-0295-0;

and Brackets in column 1 and 2 indicate changes to text made by:

Balis, L. E., Houghtaling, B., & Harden, S. M. (2022). Using implementation strategies in community settings: an introduction to the Expert Recommendations for Implementing Change (ERIC) compilation and future directions. Translational Behavioral Medicine, 12(10), 965-978. https://doi.org/10.1093/tbm/ibac061

This highlights the changes made to ERIC strategies and concepts to adapt from clinical to a community setting by those authors. Italicised text indicates where changes were made for the current study.
Column 3 and 4 highlight changes made to the ERIC concept and strategy names and definitions (developed for both original clinical and community setting) for this systematic review (in bold text).
